# Ultrasound-assisted enzymatic extraction and properties of polysaccharide from *Nostoc commune*

**DOI:** 10.1016/j.fochx.2026.104084

**Published:** 2026-06-10

**Authors:** Jinhua Shao, Chenxu Wu, Quan Li, Yuting He, Zhiyong Zhu

**Affiliations:** College of Chemistry and Bioengineering, Hunan University of Science and Engineering, Yongzhou 425199, China

**Keywords:** Ultrasound, Enzymatic method, *N. commune*, Polysaccharides, Properties

## Abstract

This study used *Nostoc commune* as the raw material to extract polysaccharides through ultrasound-assisted enzymatic extraction (UAE). A combined enzyme system consisting of pectinase, cellulase, and papain at a ratio of 1:1:1 was applied. Single-factor experiments were first conducted to evaluate the effects of solid-to-liquid ratio, enzyme dosage, enzymolysis temperature, enzymolysis time, ultrasound temperature, ultrasound power, and ultrasound duration on polysaccharide yield. Subsequently, the Plackett-Burman (PB) design was used to identify the three most significant factors affecting extraction efficiency. The steepest ascent method was then employed to determine the center point for the response surface methodology (RSM) experiments. Finally, the extraction process was optimized using the Box-Behnken (BB) design. The optimal extraction conditions were as follows: solid-to-liquid ratio of 1:80 g/mL, enzyme dosage of 0.3 g, enzymolysis temperature of 60 °C, enzymolysis time of 60 min, ultrasound power of 780 W, ultrasound time of 40 min, and ultrasound temperature of 50 °C. Under these conditions, the polysaccharide yield reached 34.11%. Structural characterization showed that polysaccharides obtained by UAE and HW displayed typical IR and NMR characteristic peaks with only minor structural differences, while revealing that those obtained by UAE had lower molecular weights, increased uronic acid content, and a similar monosaccharide composition primarily consisting of glucose, xylose, galactose, and glucuronic acid. Compared with HW, UAE significantly increased the polysaccharide yield by 16.68%, and improved antioxidant activity. These results indicate that UAE effectively improves both the extraction efficiency and bioactivity of *N. commune* polysaccharides. The findings provide technical support for their potential industrial application.

## 1 Introduction

*Nostoc commune*, commonly known as star jelly, witch's butter, or mare's eggs, belongs to the phylum Cyanophyta, family Nostocaceae, and genus *Nostoc*. It is a widely distributed cyanobacterium (Liu et al., 2019). According to the Compendium of Materia Medica, *N. commune* is described as “shaped like wood ear fungus, sweet in nature, and non-toxic, beneficial for improving eyesight and replenishing qi.” It has been used as a medicinal material since the Eastern Jin Dynasty (317–420 CE). Traditionally, it has been considered effective in treating night blindness, burns, anxiety, indigestion, and chronic fatigue (Park et al., 2008).

The polysaccharide content of *N. commune* has been reported to reach approximately 60% (Guo, 2019). However, this value is highly dependent on the pretreatment of raw materials and the extraction process used. Existing studies have shown that the optimized polysaccharide yield obtained by the traditional hot water extraction (HW) method generally ranges from 15% to 25% (Huang et al., 1998; Pan et al., 2024). Although the alkali extraction method can increase the yield to approximately 43.1% (Yang, Zhang, He, et al., 2025), polysaccharides are easily degraded and structurally damaged under strong alkaline conditions. Modern studies have demonstrated that *N. commune* polysaccharides possess multiple biological functions. These polysaccharides can scavenge reactive oxygen free radicals, regulate intestinal microecological balance, modulate lipid metabolism, and interfere with inflammatory signaling pathways (Azmi et al., 2012; Li et al., 2022). In addition, some polysaccharides have shown effects comparable to metformin in regulating blood glucose levels and may be used as adjuvant agents for diabetes treatment (Zheng et al., 2016). Meanwhile, various plant-derived polysaccharides exhibit significant inhibitory activity against common pathogenic bacteria, including *Escherichia coli*, *Staphylococcus aureus*, *Pseudomonas aeruginosa*, *Bacillus subtilis*, and *Salmonella typhimurium* (Gao et al., 2023; Han et al., 2016; Li et al., 2018). Therefore, polysaccharides are considered promising natural sources of antioxidants, antibacterial agents, and nutritional supplements because of their diverse biological activities. It is noteworthy that polysaccharides secreted by different *Nostoc* species, such as *N. flagelliforme* and *N. sphaeroides*, differ in monosaccharide composition and molecular weight (MW) distribution (Quan et al., 2015). In addition, extraction conditions such as temperature, solvent polarity, and physical field effects can alter the spatial conformation of polysaccharides. These structural changes ultimately influence their biological activities, including antioxidant and immunomodulatory properties (Hou et al., 2024a; Zhang et al., 2025). Therefore, the composition and biological activity of polysaccharides are directly affected by the raw material source, extraction method, and processing conditions (Chen et al., 2019; Dou et al., 2021; Geng et al., 2024).

Current polysaccharide extraction methods mainly include HW (Xiong et al., 2015), ultrasound-assisted extraction (UAE) (Ma et al., 2024; Wang et al., 2015), and enzymatic extraction (Chen et al., 2018). HW usually requires high temperatures and long extraction times. These conditions may alter the structure of polysaccharides and result in relatively low extraction yields (Fang & Ding, 2007). In contrast, UAE utilizes the mechanical vibration and cavitation effects generated by ultrasound in aqueous media to accelerate substance release. This method offers several advantages, including lower operating temperatures, shorter extraction times, and the absence of solvent residues. It also helps reduce the loss of active components (Wang et al., 2015). Enzymatic extraction disrupts cell walls through enzymolysis, which promotes the dissolution of bioactive compounds under mild conditions and generally results in higher recovery rates (Chen et al., 2016; Yang, Li, Wu, et al., 2025). UAE combines the physical cell wall disruption caused by ultrasound with the specific catalytic action of enzymes (Hilbig et al., 2018; Muniz-Marquez et al., 2013). This combined approach can significantly shorten extraction time, reduce energy consumption, and improve both the yield and bioactivity of polysaccharides (Bai et al., 2022; Gómez-García et al., 2012; Tang, He, et al., 2023). However, most current studies on *N. commune* polysaccharide extraction focus mainly on conventional HW or single UAE methods (Hou et al., 2024b; Huang et al., 1998). Systematic optimization of the synergistic effects of ultrasound and enzymes remains limited. Different extraction methods can produce large variations in yield, ranging from 8% to 43%. In addition, changes in extraction conditions may alter the physicochemical properties of polysaccharides, thereby affecting their structure-activity relationships. Therefore, developing efficient and mild extraction methods that preserve the active conformation of polysaccharides is of great importance. Although UAE has been widely studied for extracting intracellular substances (Qi et al., 2025; Yang, Li, Li, et al., 2025; Yi et al., 2025), its application in *N. commune* polysaccharide extraction is still at an early exploratory stage. Further systematic optimization of the relevant process parameters is urgently needed.

With the rapid development of statistical methods, their applications have expanded into fields such as biology, food science, and agriculture (Zhang, Lei, et al., 2010). Statistical experimental designs are highly valued because they can simplify workflows, reduce the number of experiments, improve product quality, and lower research costs (Zhu, 2017). The Plackett-Burman (PB) design enables the rapid identification of key factors that significantly influence a target response in complex multi-factor systems, even when the underlying mechanisms are unclear. This method requires only a limited number of experiments and effectively avoids unnecessary time and resource consumption on non-significant factors (Wang et al., 2014; Yatsyshyn et al., 2014; Zhang, Yang, et al., 2010). However, factors identified as significant by the PB design still require further optimization through response surface methodology (RSM). It should be noted that a response surface model can accurately represent real experimental conditions only within the investigated factor range. Its predictive accuracy decreases considerably outside this range (Zhu, 2017). Therefore, before conducting RSM experiments, the steepest ascent method is usually applied to approach the optimal region and determine the center point for the response surface design (Wang & Liu, 2008).

The steepest ascent design determines the direction of optimization according to the effect values of factors obtained from the PB design. Factors with positive effects are increased, whereas factors with negative effects are decreased. The step size is determined based on the magnitude of the effect values (Gou, 2014; Zhu et al., 2021). A series of experiments is then conducted along this path to evaluate how factor changes influence the response. This process helps identify the center point for subsequent response surface analysis (Dean et al., 2017). RSM can establish a quadratic polynomial model using a relatively small number of experiments. This method has several advantages, including a short experimental cycle, high regression accuracy, and the ability to evaluate interactions among factors. Therefore, it enables efficient and comprehensive optimization of experimental systems (Ni et al., 2025). The resulting quadratic model can more accurately describe the nonlinear relationships between factors and responses, thereby facilitating precise prediction of the optimal process conditions (Wen et al., 2025).

To optimize the UAE process for extracting *N. commune* polysaccharides, single-factor experiments were first conducted to preliminarily evaluate key process parameters, including solid-to-liquid ratio, enzyme dosage, enzymolysis temperature, enzymolysis time, ultrasound power, ultrasound duration, and ultrasound temperature. Based on the three significant factors identified by the PB design, the steepest ascent method was applied to approach the region of maximum response and determine the center point. Subsequently, a Box-Behnken (BB) design was employed to establish a quadratic regression model and analyze the interactions among the selected factors. Multiple regression analysis was then used to determine the optimal parameter combination for maximizing polysaccharide yield. To evaluate the advantages of UAE, this method was compared with traditional HW, including a systematic analysis of extraction yield differences. The infrared (IR) spectral characteristics of polysaccharides obtained by both methods were examined. In addition, their antioxidant activities were comprehensively evaluated through DPPH radical scavenging activity, hydroxyl radical (·OH) scavenging activity, superoxide anion radical (O_2_^−^·) scavenging activity, anti-lipid peroxidation capacity, and total reducing power assays. This study provides a comprehensive evaluation of the extraction efficiency and bioactive properties of UAE-derived *N. commune* polysaccharides. It also offers a scientific basis and practical guidance for developing efficient and environmentally friendly extraction technologies.

## 2 Materials and methods

### 2.1 Sample pretreatment

*N. commune* was collected from Yongzhou City, Hunan Province, China. The raw material was first cleaned and dried at 60 °C. It was then ground into a fine powder and passed through a 100-mesh sieve. The powder was defatted by reflux extraction using absolute ethanol at a solid-to-liquid ratio of 1:15 (g/mL) at 80 °C for 2.0 h. After extraction, the mixture was filtered, and the solid residue was collected. The residue was dried, ground again into powder, passed through a 60-mesh sieve, and stored in a sealed container for subsequent analysis.

### 2.2 Determination of *N. commune* polysaccharide content

#### 2.2.1 Preparation of glucose standard curve

The method reported by Huang et al. was adopted with slight modifications (Huang et al., 2026). A precisely weighed amount (20 mg) of glucose standard, previously dried to constant weight at 105 °C, was dissolved in distilled water and transferred to a 500 mL volumetric flask to prepare a stock solution with a concentration of 40 μg/mL. Aliquots of this standard solution (0.0, 0.4, 0.6, 0.8, 1.0, 1.2, 1.4, 1.6, and 1.8 mL) were pipetted into separate 10 mL colorimetric tubes. Distilled water was added to each tube to adjust the total volume to 2.0 mL. Subsequently, 1.0 mL of 6% phenol solution and 5.0 mL of concentrated sulfuric acid were added. The mixtures were thoroughly shaken, heated in a boiling water bath for 15 min, and then cooled to room temperature. Absorbance was measured at 490 nm. A standard calibration curve was constructed using glucose concentration as the x-axis and absorbance as the y-axis. The resulting regression equation was Y = 0.004× − 0.031, with R^2^ = 0.9998.

#### 2.2.2 Preparation of *N. commune* polysaccharides

The method proposed by Bobo Lin et al. was referenced with slight modifications (Lin et al., 2023). A 10.0 g portion of defatted *N. commune* dry powder was mixed with deionized water at a predetermined solid-to-liquid ratio. The mixture was thoroughly shaken and allowed to react under specified conditions, including composite enzyme dosage, enzymolysis temperature, and enzymolysis time. The solution was then subjected to ultrasonic extraction using a THC-type ultrasonic device under controlled conditions of ultrasound temperature, ultrasound power, and ultrasound duration. After extraction, the mixture was filtered under vacuum, and the filtrate was collected as the crude *N. commune* polysaccharide extract. The extraction yield was subsequently determined. The crude extract was concentrated to one-fifth of its original volume using a rotary evaporator. Polysaccharides were precipitated by adding 85% ethanol, followed by incubation at 4 °C for 24 h. The resulting precipitate was collected and freeze-dried under vacuum. The freeze-dried sample was redissolved in water to prepare a 5% (*w*/*v*) solution. Proteins were removed using the Sevag method (n-butanol:chloroform = 1:4). The mixture was treated at a volume ratio of 4:1 (sample to reagent, *V*/V), shaken vigorously for 20 min, and centrifuged at 4800 rpm for 10 min. This deproteinization step was repeated three additional times. The combined aqueous phases were concentrated to half of the original volume and then dialyzed using a 2000 Da molecular weight (MW) cut-off dialysis membrane. Finally, the dialyzed solution was concentrated and freeze-dried under vacuum to obtain crude *N. commune* polysaccharides.

#### 2.2.3 Determination of *N. commune* polysaccharide content

The method reported by Huang et al. was adopted with slight modifications (Huang et al., 2026). The crude polysaccharide extract was diluted 100-fold. A 1 mL aliquot of the diluted solution was transferred into a colorimetric tube and processed according to the procedure described in Section 2.2.1. The polysaccharide yield was then calculated using the glucose standard curve and the following Eq. (1):

(1) $$X\%=\frac{A\times B\times C}{m\times 10^{6}}\times 100$$

where X represents the polysaccharide extraction yield of the sample (%), A is the mass concentration of the sample solution (μg/mL), B is the total volume of the sample solution (mL), C is the dilution factor, and m is the mass of *N. commune* powder used (g).

### 2.3 HW of *N. commune* polysaccharides

This procedure was adapted from Liu Jichao (Liu et al., 2018) with modifications. A 10.0 g portion of defatted *N. commune* dry powder was mixed with deionized water at a solid-to-liquid ratio of 1:50 (g/mL) and heated at 93 °C for 190 min. The mixture was then vacuum filtered, and the filtrate was collected. The polysaccharide content was determined according to the methods described in 2.2.1, 2.2.3. The collected filtrate was concentrated to one-fifth of its original volume using a rotary evaporator. Polysaccharides were precipitated by adding 85% ethanol, followed by storage at 4 °C for 24 h. The resulting precipitate was collected and freeze-dried under vacuum. The freeze-dried sample was redissolved in water to prepare a 5% (*w*/*v*) solution. Proteins were removed using Sevag reagent (n-butanol:chloroform = 1:4). The mixture was treated at a volume ratio of 4:1 (sample to reagent, *V*/V), shaken vigorously for 20 min, and centrifuged at 4800 rpm for 10 min. This deproteinization step was repeated three times. The combined aqueous phases were concentrated to half of the original volume and then dialyzed using a 2000 Da molecular weight (MW) cut-off dialysis membrane. Finally, the dialyzed solution was concentrated and freeze-dried under vacuum to obtain crude *N. commune* polysaccharides.

### 2.4 UAE of *N. commune* polysaccharides

#### 2.4.1 Single-factor experiments for UAE

A 10.0 g portion of defatted *N. commune* dry powder was used for each experiment. The general procedure followed that described in Section 2.2.2. The aim was to evaluate the effects of individual experimental conditions on polysaccharide extraction yield and to determine the optimal level of each parameter. These optimal values were subsequently used as fixed conditions in the following experiments. The specific parameters and their tested ranges were as follows: solid-to-liquid ratio (1:30, 1:40, 1:50, 1:60, 1:70 g/mL); composite enzyme dosage (0.15, 0.20, 0.25, 0.30, 0.35 g); enzymolysis temperature (30, 40, 50, 60, 70 °C); enzymolysis time (30, 40, 50, 60, 70 min); ultrasound temperature (50, 55, 60, 65, 70 °C); ultrasound power (600, 690, 780, 870, 960 W); and ultrasound time (20, 30, 40, 50, 60 min). All experiments were performed in triplicate, and the mean values were recorded for analysis.

#### 2.4.2 Plackett-Burman design

Based on the results of the single-factor experiments, Design-Expert 8.0.6 software was used to perform a PB design and analyze the data. This design was applied to evaluate seven factors potentially affecting the extraction yield of *N. commune* polysaccharides: ultrasound time (A), ultrasound temperature (B), ultrasound power (C), solid-to-liquid ratio (D), enzymolysis time (E), enzymolysis temperature (F), and enzyme dosage (G). A Pareto chart was used to identify statistically significant factors and to determine whether their effects were positive or negative. For each factor, two levels (high and low) were selected based on the results of the single-factor experiments. The polysaccharide extraction yield was used as the response variable, and the PB design was then implemented. All experiments were performed in triplicate, and the mean values were used for analysis. The specific factors and their corresponding levels are summarized in Table 1.

**Table 1:** Plackett-Burman design and results.

Exp. No.	A	B	C	D	E	F	G	Extraction rate/%
1	30(−1)	45(−1)	690(−1)	50(−1)	50(−1)	50(−1)	0.25(−1)	20.38 ± 0.12
2	50(1)	45(−1)	870(1)	70(1)	50(−1)	70(1)	0.35(1)	30.12 ± 0.18
3	30(−1)	55(1)	870(1)	50(−1)	70(1)	70(1)	0.35(1)	18.95 ± 0.16
4	50(1)	55(1)	690(−1)	70(1)	70(1)	70(1)	0.25(−1)	29.8 ± 0.15
5	50(1)	45(−1)	870(1)	70(1)	70(1)	50(−1)	0.25(−1)	27.2 ± 0.24
6	30(−1)	55(1)	870(1)	70(1)	50(−1)	50(−1)	0.25(−1)	25.41 ± 0.31
7	50(1)	45(−1)	690(−1)	50(−1)	70(1)	50(−1)	0.35(1)	23.7 ± 0.19
8	30(−1)	55(1)	690(−1)	70(1)	70(1)	50(−1)	0.35(1)	30.41 ± 0.22
9	50(1)	55(1)	690(−1)	50(−1)	50(−1)	70(1)	0.25(−1)	17.39 ± 0.36
10	50(1)	55(1)	870(1)	50(−1)	50(−1)	50(−1)	0.35(1)	20.06 ± 0.28
11	30(−1)	45(−1)	690(−1)	70(1)	50(−1)	70(1)	0.35(1)	31.99 ± 0.29
12	30(−1)	45(−1)	870(1)	50(−1)	70(1)	70(1)	0.25(−1)	15.00 ± 0.24

#### 2.4.3 Steepest ascent design

The PB design identified ultrasound power, solid-to-liquid ratio, and enzyme dosage as the three most significant factors. After determining whether each factor had a positive or negative effect, the direction and step size of the steepest ascent path were established based on their regression coefficients (Liu, Wang, et al., 2023; Lu et al., 2020). The steepest ascent path indicates the direction in which the response increases most rapidly, and the step size is proportional to the magnitude of the regression coefficients. The factor level combination that produced the highest response in the steepest ascent experiment was selected as the center point for the subsequent BB design. The specific factors, levels, and results of the steepest ascent experiments are presented in Table 3. All experiments were performed in triplicate, and the mean values were used for analysis.

#### 2.4.4 BB central composite design

RSM was used to evaluate the interaction effects among the significant factors. Using the optimal point identified through the steepest ascent design as the center, a CCD(Central Composite Design)was employed. The three significant factors identified by the PB design were selected as independent variables, and polysaccharide extraction yield was used as the response variable. All experiments were performed in triplicate, and the mean values were used for analysis.

### 2.5 MW of polysaccharides

The MW of polysaccharides was determined by high-performance gel permeation chromatography (HPGPC) with minor modifications (Liu, Zhang, et al., 2023). A calibration curve was established using polyethylene glycol (PEG) standards of different molecular weights. Exactly 20 mg of sample was weighed and transferred into a 10 mL volumetric flask. The sample was dissolved in 0.1 mol/L NaNO_3_ aqueous solution and diluted to volume. After complete dissolution for 6.0 h, the solution was filtered through a 0.22 μm microporous membrane. A 1.5 mL aliquot of the filtrate was then transferred into a 2 mL sample vial for subsequent analysis.

Instrument analysis was performed using an Agilent 1260 high-performance liquid chromatograph. The mobile phase consisted of 0.1 mol/L NaNO_3_ aqueous solution at a flow rate of 1.0 mL/min. The column temperature was maintained at 40 °C. Detection was carried out using an Agilent G1362A refractive index detector (RID). Waters Ultrahydrogel 500, 250, and 120 columns (300 × 7.8 mm) were connected in series. The injection volume was 40 μL, and the total run time was 40 min per analysis. A calibration curve was constructed based on the elution times of PEG standard solutions. The elution time of the polysaccharide samples was then applied to the standard curve, and the relative MW was calculated using Agilent GPC software.

### 2.6 Monosaccharide composition

The pre-column derivatization method using 1-phenyl-3-methyl-5-pyrazolone (PMP) was adopted (Wang, Gao, et al., 2018). Thirteen monosaccharide standards and the samples were hydrolyzed with trifluoroacetic acid (TFA), followed by derivatization with PMP. The aqueous phase was then filtered through a 0.22 μm membrane and subjected to high-performance liquid chromatography (HPLC) analysis. The chromatographic conditions were as follows: Shim-pack GIST column (5 μm, 4.6 × 150 mm), flow rate of 1.0 mL/min, column temperature of 30 °C, and detection wavelength of 254 nm. Mobile phase A consisted of 83% phosphate solution, and mobile phase B consisted of 17% acetonitrile solution. Gradient elution was applied for separation.

### 2.7 IR spectroscopy and nuclear magnetic resonance analysis

For IR spectroscopy, approximately 200 mg of potassium bromide (KBr) was dried in an oven at 110 °C for 4 h. The dried KBr was thoroughly mixed and ground with 2 mg of sample, and the mixture was pressed into a pellet. IR spectra were recorded over a scanning range of 4000 cm^−1^ with a resolution of 4 cm^−1^. For nuclear magnetic resonance (NMR) analysis, approximately 0.05 g of *N. commune* polysaccharide was dissolved in 0.5 mL of D₂O (99.99%) and transferred into an NMR tube. ^13^C NMR spectra were acquired using a 400 MHz NMR spectrometer (Lin et al., 2023).

### 2.8 In vitro antioxidant activity of *N. commune* polysaccharides

#### 2.8.1 Determination of DPPH radical scavenging capacity

The assay was performed according to the method of Liu Jianwei et al. (Wang et al., 2024), with minor modifications. Polysaccharide solutions were prepared at different concentrations (0.2, 0.4, 0.8, 1.6, and 3.2 mg/mL). A 1 mL aliquot of each sample solution was mixed with an equal volume of 0.2 mmol/L DPPH solution in ethanol. The mixtures were incubated in the dark at room temperature for 30 min, and the absorbance (A₀) was measured at 517 nm. For the control group, the DPPH solution was replaced with anhydrous ethanol, and the absorbance (A₁) was recorded. For the blank group, the sample solution was replaced with deionized water, and the absorbance (A₂) was measured. Different concentrations of vitamin C (Vc) solution were used as a positive control. All measurements were performed in triplicate, and the mean values were calculated. The DPPH radical scavenging activity was calculated using the following equation:

$$\text{DPPH Radical Scavenging Rate}\;\left(\%\right)=\left(1-\frac{A0-A1}{A2}\right)\times 100\%$$

where A_0_ is the absorbance of the sample-DPPH mixture, A_1_ is the absorbance measured when anhydrous ethanol replaces the DPPH-ethanol solution (control), and A_2_ is the absorbance of the blank control (deionized water with DPPH solution).

#### 2.8.2 Determination of ·OH scavenging capacity

The assay was performed according to the method of Wang Li et al. (Gu et al., 2023), with minor modifications. Polysaccharide solutions were prepared at different concentrations (0.2, 0.4, 0.8, 1.6, and 3.2 mg/mL). A 1 mL aliquot of each sample solution was sequentially mixed with 1 mL of 8 mmol/L FeSO_4_ solution and 1 mL of 8 mmol/L H_2_O_2_ solution. The mixtures were allowed to react at room temperature for 10 min. Subsequently, 1 mL of 8 mmol/L salicylic acid in ethanol was added. After thorough mixing, the reaction system was incubated in a 37 °C water bath for 30 min, and the absorbance (A_q_) was measured at 510 nm. For the background control, H_2_O_2_ was replaced with deionized water, and the absorbance (A_r_) was recorded. For the blank control, the polysaccharide solution was replaced with deionized water, and the absorbance (A_0_) was measured. V_c_ was used as a positive control. All measurements were performed in triplicate, and the mean values were calculated. The ·OH scavenging activity was calculated using the following equation:

$$\text{Hydroxyl Radical Scavenging Rate}\;\left(\%\right)=\left(1-\frac{\mathrm{AQ}-\mathrm{Ar}}{A0}\right)\times 100\%$$

where A_q_ is the absorbance of the sample group, A_r_ is the absorbance measured when deionized water replaces H_2_O_2_ (background control), and A_0_ is the absorbance measured when deionized water replaces the polysaccharide solution.

#### 2.8.3 Determination of O^2−^· scavenging capacity

The assay was performed according to the method of Ren Wei et al. (Zhang et al., 2024), with minor modifications. Polysaccharide solutions were prepared at different concentrations (0.2, 0.4, 0.8, 1.6, and 3.2 mg/mL). A 1 mL aliquot of each sample solution was mixed with 4 mL of 0.05 mol/L Tris-HCl buffer (pH 8.2). The mixtures were pre-incubated in a 25 °C water bath for 20 min. Subsequently, 20 μL of 3 × 10^−3^ mol/L pyrogallol solution was added. The reaction mixtures were immediately shaken and incubated at 25 °C for 5 min. The reaction was terminated by adding 200 μL of 10% HCl solution, and the absorbance (A_1_) was measured at 325 nm. For the control group, pyrogallol was replaced with an equal volume of deionized water, and the absorbance (A_2_) was recorded. For the blank group, the polysaccharide solution was replaced with deionized water, and the absorbance (A_0_) was measured. Vc was used as a positive control. All measurements were performed in triplicate, and the mean values were calculated. The O^2−^· scavenging activity was calculated using the following equation:

$$\text{Superoxide Anion Radical Scavenging Rate}\;\left(\%\right)=\left(1-\frac{A1-A2}{A0}\right)\times 100\%$$

where A_1_ is the absorbance of the sample group, A_2_ is the absorbance measured when deionized water replaces the pyrogallol solution, and A_0_ is the absorbance measured when deionized water replaces the polysaccharide sample.

#### 2.8.4 Determination of anti-lipid peroxidation capacity

The assay was performed according to the method of Bobo L et al. (Lin et al., 2023), with minor modifications. Polysaccharide solutions were prepared at different concentrations (0.2, 0.4, 0.8, 1.6, and 3.2 mg/mL). A 1 mL aliquot of each solution was mixed with 1 mL of 20% trichloroacetic acid and 1 mL of 0.8% thiobarbituric acid. After thorough mixing, the tubes were heated in a boiling water bath for 15 min and then cooled to room temperature. The mixtures were centrifuged at 3600 rpm for 10 min, and the supernatants were collected. The absorbance (A_1_) was measured at 535 nm. For the blank control, the polysaccharide solution was replaced with an equal volume of deionized water, and the absorbance (A_0_) was recorded. Vc was used as a positive control. All measurements were performed in triplicate, and the mean values were calculated. The anti-lipid peroxidation capacity was calculated using the following equation:

$$E\left(\%\right)=\frac{A0-A1}{A0}\times 100\%$$

where A_0_ is the absorbance measured when deionized water replaces the sample solution, and A_1_ is the absorbance of the sample solution.

#### 2.8.5 Determination of total reducing power

The assay was performed according to the method of Wang Ping et al. (Wang et al., 2024), with minor modifications. Polysaccharide solutions were prepared at different concentrations (0.2, 0.4, 0.8, 1.6, and 3.2 mg/mL). A 2 mL aliquot of each solution was mixed with 2 mL of phosphate buffer (0.2 mol/L, pH 7.4) and 2 mL of 1% potassium ferricyanide solution. The mixtures were incubated in a 50 °C water bath for 20 min, then rapidly cooled to room temperature. Subsequently, 2 mL of 10% trichloroacetic acid was added to terminate the reaction. The mixtures were centrifuged at 3000 rpm for 10 min, and 2.5 mL of the supernatant was collected and mixed with 0.5 mL of 0.1% FeCl_3_ solution. After standing for 10 min, the absorbance was measured at 700 nm. Higher absorbance values indicate stronger reducing power. Vc was used as a positive control. All measurements were performed in triplicate, and the mean values were calculated. The total reducing power was determined using the following equation:

$$A=A_{1}-A_{2}$$

where A_1_ is the absorbance of the sample group, and A_2_ is the absorbance measured when PBS buffer replaces the polysaccharide sample.

## 3 Results and discussion

### 3.1 Influence of different factors on the extraction yield of *N. commune* polysaccharides

**Fig. 1:**
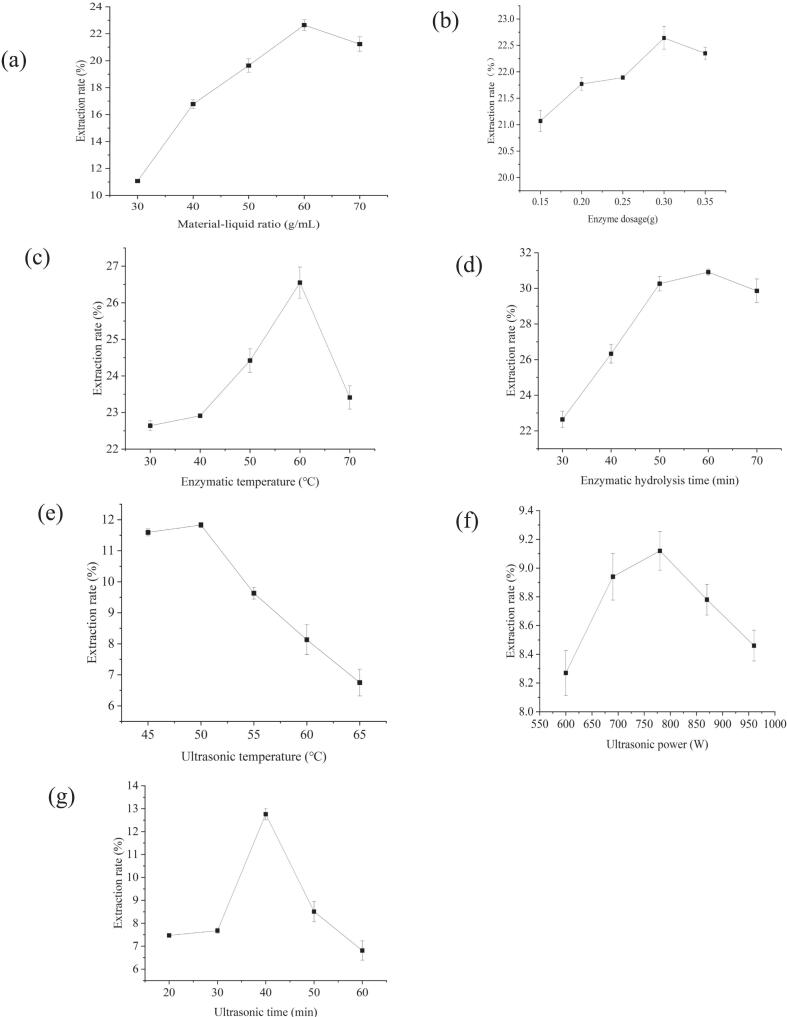
Effects of different factors on the extraction yield of *N. commune* polysaccharides; a: Solid-to-liquid ratio; b: Enzyme dosage; c: Enzymolysis temperature; d: Enzymolysis time; e: Ultrasound temperature; f: Ultrasound power; g: Ultrasound time.

As shown in Fig. 1a, the polysaccharide extraction yield first increased and then decreased as the solid-to-liquid ratio varied from 1:30 to 1:70 g/mL, reaching a maximum at 1:60 g/mL. This trend can be explained by enzyme-substrate interactions. At a low solid-to-liquid ratio, the substrate concentration is excessively high, which can saturate enzyme molecules. As a result, active sites become occupied, reducing effective enzyme-substrate binding and lowering reaction efficiency (Gu et al., 2023). In contrast, at a high solid-to-liquid ratio, the substrate concentration is too low. Although enzyme active sites are available, insufficient substrate limits catalytic activity (Gu et al., 2023). Therefore, 1:60 g/mL was selected as the optimal solid-to-liquid ratio.

The effect of enzyme dosage on polysaccharide extraction yield is shown in Fig. 1b. As the enzyme dosage increased from 0.15 to 0.35 g, the yield first increased and then decreased, reaching a maximum at 0.30 g. An appropriate enzyme dosage enhances substrate breakdown and promotes polysaccharide release. However, excessive enzyme (>0.30 g) leads to a saturation effect, where additional enzyme molecules no longer contribute effectively to catalysis and have little impact on yield improvement. These findings are consistent with those reported by Zhang Zhanxia et al. (Zhang et al., 2024) Therefore, 0.30 g was selected as the optimal enzyme dosage.

Fig. 1c illustrates the effect of enzymolysis temperature on polysaccharide extraction yield. Within the tested range of 30–70 °C, the yield first increased and then decreased, reaching a maximum at 60 °C. Enzyme activity generally increases with temperature, which enhances catalytic efficiency. However, temperatures above the optimal range can cause thermal denaturation and irreversible inactivation of the enzyme, leading to a reduction in extraction yield (Saeed & Ahmed, 2024). Therefore, 60 °C was selected as the optimal enzymolysis temperature.

As shown in Fig. 1d, the extraction yield increased with enzymolysis time and reached a maximum at 60 min, after which it remained stable. This indicates that substrate release and polysaccharide dissolution reached equilibrium within this period. Extending the reaction time beyond 60 min did not further improve the yield and may even lead to polysaccharide degradation or other unfavorable side reactions, thereby reducing extraction efficiency (Dong et al., 2015). Therefore, 60 min was selected as the optimal enzymolysis time.

Fig. 1e shows that increasing ultrasound temperature caused the polysaccharide extraction yield to first increase and then decrease, with a maximum at 50 °C. At lower temperatures, the cavitation effect generated by ultrasound is relatively weak and provides insufficient energy to effectively disrupt *N. commune* cell walls and release polysaccharides. In contrast, excessively high temperatures can lead to degradation of polysaccharide structures and inactivation of heat-sensitive bioactive components (Aslam et al., 2023). Although moderate heating can enhance enzymatic hydrolysis, temperatures above the enzyme's optimal range are unfavorable. Since enzymolysis was conducted separately at 60 °C, maintaining the ultrasound temperature at 50 °C also helps prevent premature inactivation of enzyme proteins (Saeed & Ahmed, 2024). Therefore, 50 °C was selected as the optimal ultrasound temperature.

Fig. 1f shows that the extraction yield increased with ultrasound power, reaching a maximum at 780 W, and then decreased at higher power levels. At low ultrasound power, cavitation and mechanical effects are insufficient to effectively disrupt cell walls, resulting in limited polysaccharide release. In contrast, excessively high power generates localized high temperatures, which can lead to polysaccharide degradation and reduced bioactivity. High-power ultrasound may also produce excessive foaming, which destabilizes the reaction system (Bahadar et al., 2015). Therefore, 780 W was selected as the optimal ultrasound power.

Fig. 1g illustrates the effect of ultrasound duration on polysaccharide extraction yield. Within the tested range of 20–60 min, the yield first increased, reached a maximum at 40 min, and then decreased. An appropriate ultrasound duration (≤40 min) enhances cavitation and mechanical effects. This effectively disrupts cellular structures and promotes the release of intracellular polysaccharides and enzymatic components (Shen et al., 2023). However, prolonged ultrasonication (>40 min) can produce several adverse effects. These include breakage of peptide chains and disruption of disulfide bonds in enzyme molecules, which reduces catalytic activity. Continuous energy input may also cause excessive temperature rise, leading to substrate degradation and enzyme aggregation. In addition, extended ultrasonication can increase impurity levels in the crude extract, making downstream separation and purification more difficult. Therefore, 40 min was selected as the optimal ultrasound time.

### 3.2 Plackett-Burman design resultss

Based on the results of the single-factor experiments, a PB design was conducted using Design-Expert 8.0.6 software. The variables were defined as follows: A, ultrasound time (min); B, ultrasound temperature (°C); C, ultrasound power (W); D, solid-to-liquid ratio (g/mL); E, enzymolysis time (min); F, enzymolysis temperature (°C); and G, enzyme dosage (g). The PB design and corresponding results are summarized in Table 1, while the analysis of variance (ANOVA) is presented in Table 2.

**Table 2:** ANOVA of the Plackett-Burman.

Project	sum of squares	freedom	mean square	F-value	*P*-value	Significance
Model	359.70	7	51.39	29.47	0.0028	**
A	3.13	1	3.13	1.80	0.2513	
B	3.38	1	3.38	1.94	0.2362	
C	23.89	1	23.89	13.70	0.0208	*
D	294.53	1	294.53	168.91	0.0002	**
E	7.008E-003	1	7.008E-003	4.019E-003	0.9525	
F	1.27	1	1.27	0.73	0.4408	
G	33.50	1	33.50	19.21	0.0118	*
Residual	6.97	4	1.74			
Cor Total	366.68	11				

Note: * indicates a significant difference (P < 0.05); ** indicates a highly significant difference (P < 0.01); R^2^ = 0.9810, R^2^Adj = 0.9477; CV = 5.46%; Adeq Precisio = 14.951.

As shown in Table 2, the PB model was statistically significant (*p* = 0.0028) and accounted for 94.77% of the response variation (adjusted R^2^ = 0.9477). The model also showed good reliability and precision, as indicated by a coefficient of variation (CV) of 5.46% and an adequate precision value of 14.951. The effects of the investigated factors on polysaccharide extraction yield were ranked as follows: solid-to-liquid ratio (D) > enzyme dosage (G) > ultrasound power (C). Among them, factors A, B, E, and F showed no significant effects within the studied range, whereas D, C, and G exhibited significant or highly significant effects (*p* < 0.05 or *p* < 0.01). Therefore, these three factors were selected as key variables for subsequent steepest ascent experiments and BB optimization.

### 3.3 Analysis of the path of steepest ascent

PB experiments showed that the solid-to-liquid ratio (D) and enzyme dosage (G) had positive effects on polysaccharide extraction yield, whereas ultrasound power (C) exerted a negative effect. This may be related to polysaccharide degradation induced by cavitation and shear forces under high ultrasound power (Guo et al., 2025a). The direction and step size of the steepest ascent were determined based on the coefficient values of the significant factors identified in the PB experiments (Liu, Wang, et al., 2023; Lu et al., 2020). As shown in Table 3, the highest polysaccharide yield was obtained in the second experimental group (enzyme dosage 0.32 g, solid-to-liquid ratio 1:70 g/mL, ultrasound power 750 W). This condition was considered close to the optimal region and was therefore selected as the center point for subsequent CCD optimization.

**Table 3:** Design and results of the steepest ascent experiment.

Exp. No.	C/W	D/g/mL	G/g	Extraction rate/%
1	780	60	0.3	32.55 ± 0.08
2	750	70	0.32	33.63 ± 0.17
3	720	80	0.34	32.89 ± 0.11
4	690	90	0.36	30.38 ± 0.14
5	660	100	0.38	30.15 ± 0.19
6	630	110	0.40	30.04 ± 0.13

### 3.4 CCD and RSM

Based on the results of the PB and steepest ascent designs, a CCD response surface optimization experiment was conducted. The three significant factors, A (ultrasound power, W), B (solid-to-liquid ratio, g/mL), and C (enzyme dosage, g), were selected as independent variables, with polysaccharide extraction yield as the response variable. The coded levels of these factors are presented in Table 4. A three-factor, three-level CCD was performed using Design-Expert 8.0 software, and the experimental design and results are summarized in Table 5.

**Table 4:** Factors and levels in central composite design.

Factors	Levels		
	−1	0	1
A: Ultrasound power	720	750	780
B: Solid-to-liquid ratio	60	70	80
C: Composite enzyme dosage	0.3	0.32	0.34

**Table 5:** Central composite design layout and experimental results.

Exp. No.	A/W	B/g/mL	C/g	Extraction rate/%
1	720	70	0.3	32.11 ± 0.51
2	720	60	0.32	31.31 ± 0.43
3	750	70	0.32	32.61 ± 0.48
4	750	60	0.3	31.77 ± 0.37
5	750	80	0.3	33.93 ± 0.29
6	750	70	0.32	32.63 ± 0.41
7	720	80	0.32	33.49 ± 0.73
8	750	70	0.32	32.46 ± 0.69
9	780	70	0.34	32.13 ± 0.55
10	780	70	0.3	32.77 ± 0.63
11	750	80	0.34	33.65 ± 0.19
12	750	70	0.32	32.62 ± 0.24
13	780	60	0.32	31.78 ± 0.83
14	720	70	0.34	32.19 ± 0.56
15	750	70	0.32	32.59 ± 0.61
16	750	60	0.34	31.61 ± 0.74
17	780	80	0.32	33.71 ± 0.68

Analysis of the BB results in Table 5 yielded the following regression model for polysaccharide extraction yield: Y = 32.58 + 0.16 A + 1.04B − 0.13C − 0.063AB − 0.18 AC − 0.03 BCE − 0.22A^2^ + 0.22B^2^ − 0.057C^2^. ANOVA (Table 6) showed that the regression model was highly significant (*p* < 0.0001), while the lack-of-fit term was not significant (*p* > 0.05). The coefficient of determination (R^2^) was 0.9976, the adjusted R^2^ was 0.9944, and the CV was 0.18%, indicating excellent model fitting and strong predictive reliability. Among the model terms, A, B, C, the interaction term AC, and the quadratic terms A^2^ and B^2^ were statistically significant (p < 0.01). The negative coefficient of AC indicated a negative interaction between ultrasound power and enzyme dosage, suggesting that simultaneously high levels of both factors were unfavorable for polysaccharide extraction. This may be attributed to enzyme denaturation caused by localized high temperature and pressure generated during intense ultrasonic cavitation, which reduces enzymatic catalytic efficiency (Kadkhodaee & Povey, 2008; O'Donnell et al., 2010). In addition, the negative coefficient of A^2^ indicated an inverted U-shaped relationship between ultrasound power and extraction yield, suggesting the existence of an optimal ultrasound intensity. The positive coefficient of B^2^ indicated a U-shaped relationship between solid-to-liquid ratio and extraction yield, implying lower yields at intermediate levels and higher yields at both low and high levels. This behavior may be related to the balance between mass transfer driving force and solid-liquid contact efficiency under different conditions (Bezerra et al., 2008).

**Table 6:** ANOVA for the regression model.

Project	sum of squares	freedom	mean square	F-value	P-value	Significance
Model	9.52	9	1.06	317.57	<0.0001	**
A	0.21	1	0.21	62.48	<0.0001	**
B	8.63	1	8.63	2592.75	<0.0001	**
C	0.13	1	0.13	37.55	0.0005	**
AB	0.016	1	0.016	4.69	0.0670	
AC	0.13	1	0.13	38.93	0.0004	**
BC	3.600E-003	1	3.600E-003	1.08	0.3330	3.600E-003
A^2^	0.21	1	0.21	63.88	<0.0001	**
B^2^	0.20	1	0.20	58.60	0.0001	**
C^2^	0.014	1	0.014	4.15	0.0812	
Residuals	0.023	7	3.329E-003			
Lack-of-fit error	3.825E-003	3	1.275E-003	0.26	0.8501	
Pure error	0.019	4	4.870E-003			
Sum	9.54	16				

Note: * indicates a significant difference (*P* < 0.05); ** indicates a highly significant difference (*P* < 0.01).

**Fig. 2:**
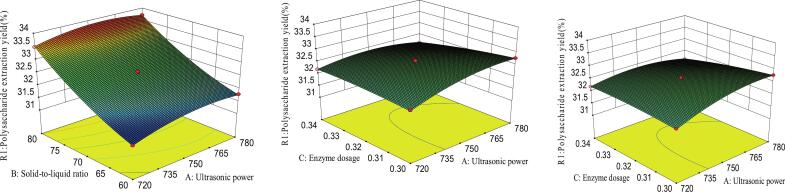
3D response surface plots showing the interactive effects of different factors on the extraction yield of *N. commune* polysaccharides.

The 3D response surface plots (Fig. 2) further supported the above findings. The response surface of the A–C interaction showed a steep gradient, indicating a significant interaction between ultrasound power and enzyme dosage on polysaccharide extraction yield. In contrast, the response surfaces of A–B and B–C interactions were relatively flat, suggesting weak or insignificant interaction effects. These observations are consistent with the ANOVA results.

### 3.5 Model validation and diagnostic analysis

**Fig. 3:**
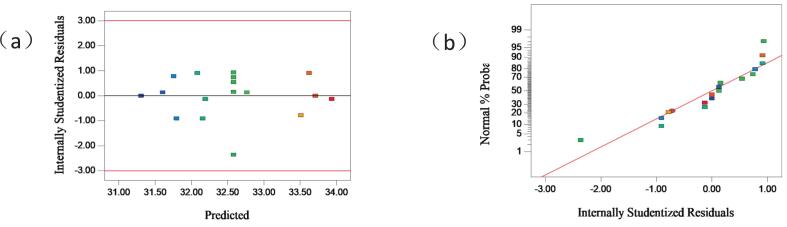
Model diagnostic plots.

Residual analysis was used to evaluate the adequacy of the established model. As shown in Fig. 3(a), the internally studentized residuals were randomly scattered, indicating that the variance of the residuals was homogeneous. In addition, the residuals followed an approximately normal distribution, as shown in Fig. 3(b). This confirms that the assumption of normality was satisfied. Overall, only small deviations were observed between the experimental and predicted values, indicating good model fitting accuracy and strong practical applicability (Li et al., 2017).

### 3.6 Verification experiment

The response surface optimization predicted the optimal extraction conditions as follows: ultrasound power of 768.63 W, solid-to-liquid ratio of 1:80 g/mL, and enzyme dosage of 0.30 g, with a corresponding predicted polysaccharide yield of 34.02%. For practical application, these conditions were slightly adjusted to an ultrasound power of 780 W, a solid-to-liquid ratio of 1:80 g/mL, and an enzyme dosage of 0.30 g. Under these adjusted conditions, the actual polysaccharide yield was 34.11% ± 0.37%. The small deviation between the predicted and experimental values confirms the reliability of the model and demonstrates the feasibility and robustness of the optimized extraction process.

### 3.7 Influence of different extraction methods on the extraction yield of *N. commune* polysaccharides

To validate the effectiveness of the proposed method, a comparative experiment was conducted using a reference method. According to the HW procedure optimized by Liu Jichao (Liu et al., 2018), 1.0 g of defatted *N. commune* dry powder was extracted with deionized water at a solid-to-liquid ratio of 1:50 (g/mL) and a temperature of 363.15 K for 180 min. After vacuum filtration, the polysaccharide yield was determined as described in Section 2.2.3, resulting in 17.43% ± 0.17% (mean of three replicates). In contrast, the UAE method developed in this study achieved a yield of 34.11% ± 0.37%, nearly twice that of Liu's HW method, demonstrating a substantial improvement. Further comparison with the enzymatic extraction method reported by Zhang Zhanxia (Zhang et al., 2024) (24.69%) and the UAE method reported by Li Haiping (Li et al., 2013) (22.59%) confirmed that the proposed UAE approach consistently produces higher yields. These results indicate that the UAE method, through the synergistic effects of ultrasound and enzymatic hydrolysis, significantly enhances the extraction efficiency of *N. commune* polysaccharides and demonstrates strong potential for practical application.

### 3.8 MW distribution of polysaccharides

The MW parameters of *N. commune* polysaccharides obtained by HW and UAE are presented in Table 7. Both extracts exhibited a bimodal distribution. HW preserved the native macromolecular structure of the polysaccharides. Its primary weight-average Mw reached 123,089 Da, and the peak molecular weight (Mp) was 176,405 Da, which was consistent with the high-molecular-weight feature of natural plant polysaccharides (Xu et al., 2023). A secondary pe ak with a much lower Mw (5141 Da) was also detected, likely representing smaller fragments or oligosaccharides natively present in the matrix. However, a relatively high polydispersity index (PDI) of 3.07 for the primary peak indicated a broad MW distribution and heterogeneous composition in the crude extract.

**Table 7:** Determination results of polysaccharide molecular weight.

Sample name	Peak molecular weight (Mp)/Da	Number-average molecular weight (Mn)/Da	Weight-average molecular weight (Mw)/Da	*Z*-average molecular weight (Mz)/Da	Polydispersity index (PDI)
HW	176,405	40,030	123,089	287,978	3.07
	7713	4020	5141	6327	1.28
UAE	24,362	15,086	35,093	81,498	2.33
	4245	3022	3277	3536	1.08

In contrast, UAE significantly altered the MW distribution of both fractions. After this treatment, the primary Mw of polysaccharides dropped sharply to 35,093 Da, the corresponding Mp decreased to 24,362 Da, and PDI was reduced from 3.07 to 2.33. Similarly, the Mw of the secondary peak decreased to 3277 Da with a narrow PDI of 1.08. This pronounced depolymerization and homogenization resulted from the synergistic effects of ultrasonic physical fields and enzymatic hydrolysis. On the one hand, microjets and strong shear force were generated by ultrasonic cavitation in liquid systems. Polysaccharide molecular chains were physically broken, and the structure of plant cell walls and matrix was destroyed, so that the mass transfer rate was greatly improved (Guo et al., 2025b; Zhong et al., 2015). On the other hand, the exposed polysaccharide chains were more easily recognized by specific enzymes. The depolymerization of macromolecules was further accelerated by the specific hydrolysis of glycosidic bonds (Prajapat et al., 2016). The coupling mechanism of “physical fragmentation-biological enzymatic cleavage” not only removed insoluble ultra-high MW components effectively, but also made the MW distribution converge toward low molecular weight, and the sample homogeneity was significantly improved accompanied by the decrease of PDI. It has been reported that moderately degraded low-molecular-weight polysaccharides generally exhibit better solubility, improved rheological properties, and enhanced antioxidant activity (Fan et al., 2025; Zeng et al., 2023).

### 3.9 Analysis of monosaccharide composition

**Fig. 4:**
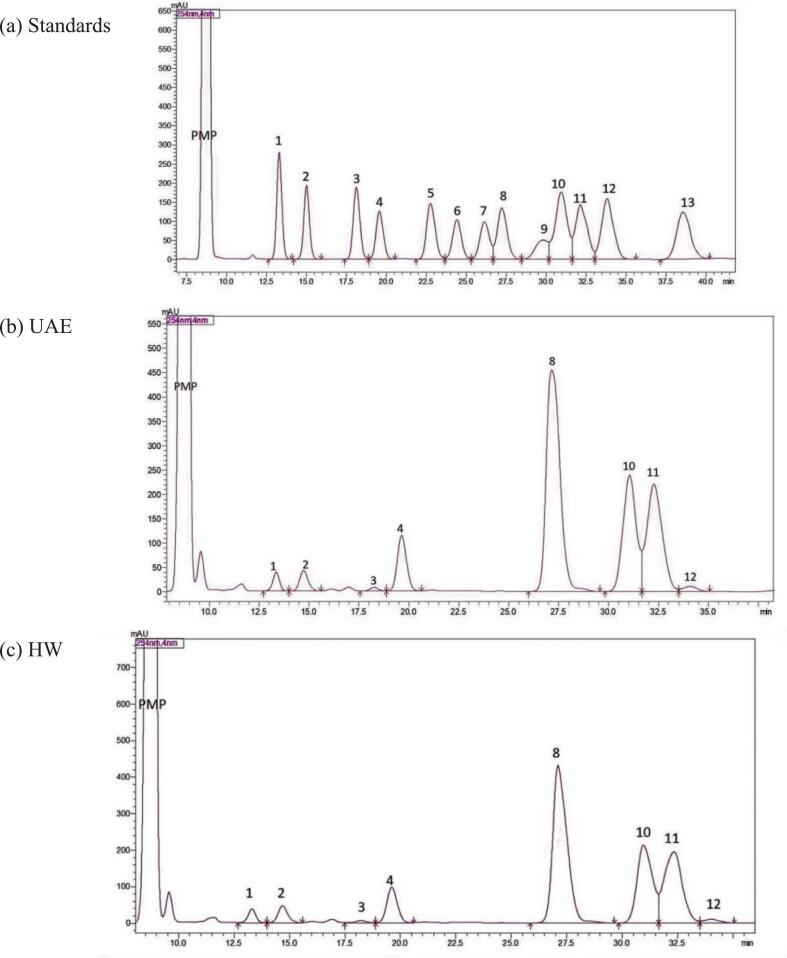
HPLC chromatograms of monosaccharides after PMP derivatization; PMP: derivatization reagent; 1. Mannose; 2. Glucosamine; 3. Rhamnose; 4. Glucuronic acid; 5. Galacturonic acid; 6. Galactosamine; 7. *N*-acetylglucosamine; 8. Glucose; 9. *N*-acetylgalactosamine; 10. Galactose; 11. Xylose; 12. Arabinose; 13. Fucose.

As shown in the monosaccharide composition results (Fig. 4), nearly identical monosaccharide profiles were observed for polysaccharides extracted by UAE and HW methods. The main constituents were glucose (45.29%, 43.86%), xylose (23.31%, 24.09%), galactose (14.56%, 14.74%), and glucuronic acid (10.36%, 9.87%), which together accounted for more than 90% of the total monosaccharides. Minor variations in monosaccharide proportions were attributed to the synergistic effects of ultrasonic cavitation-induced shear forces and enzymatic hydrolysis (Kabawa et al., 2026). Ultrasonic treatment exposes enzyme-accessible cleavage sites on polysaccharide chains, while the collapse of cavitation bubbles generates shear forces that promote moderate degradation of both main chains and side chains (Yuan et al., 2022). Different regions of polysaccharides, including backbone and side chains, exhibit varying sensitivities to physical shear and enzymatic hydrolysis due to differences in glycosidic bond types and steric hindrance. This results in differential chain scission patterns and, consequently, slight variations in the final monosaccharide molar ratios (Cheung et al., 2018; Nachtigall et al., 2021). Notably, both extraction methods yielded relatively high glucuronic acid contents (approximately 10%), while galacturonic acid was not detected. This indicates that the polysaccharides are acidic heteropolysaccharides in which glucuronic acid is the main acidic component. The glucuronic acid content was slightly higher in UAE-extracted polysaccharides, which is generally associated with improved antioxidant activity. It has been reported that uronic acid content is a key determinant of the in vitro antioxidant capacity of polysaccharides (Pan et al., 2024), and samples enriched in uronic acid residues typically exhibit stronger antioxidant activity (Su & Li, 2020a; Zhang et al., 2013).

### 3.10 IR spectroscopy

**Fig. 5:**
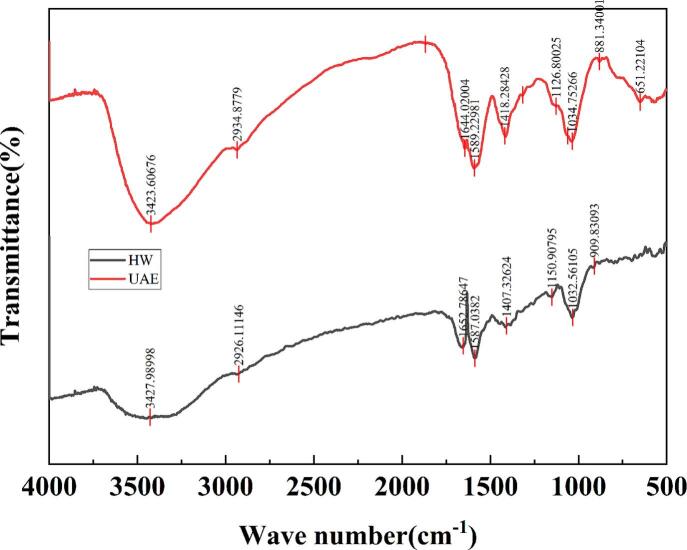
FT-IR absorption spectra of *N. commune* polysaccharides.

According to FTIR analysis (Fig. 5), both UAE and HW polysaccharides exhibited broad and strong absorption peaks at 3423.6 cm^−1^ and 3427.9 cm^−1^, respectively, which were attributed to O—H stretching vibrations (Wei & Zhang, 2023). Compared with the HW sample, a slight blue shift of the O—H peak was observed in the UAE sample, indicating weakened hydrogen bonding interactions (Wang, Chen, et al., 2018). This phenomenon may be caused by partial degradation of polysaccharide chains induced by ultrasonic cavitation (Joseph & Jemmis, 2007). Weak absorption peaks at 2934.8 cm^−1^ (UAE) and 2926.1 cm^−1^ (HW) were assigned to asymmetric C—H stretching vibrations. A slight red shift of the C—H peak was observed in the UAE sample. This may be attributed to the exposure of more terminal —CH_2_— groups following chain scission. Compared with the long, coiled polysaccharide chains extracted by HW, short-chain fragments generated by ultrasonic degradation expose more terminal methylene groups, thereby altering the electron cloud environment of C—H bonds (Yuen et al., 2009). Absorption bands at 1644.0 cm^−1^, 1652.7 cm^−1^, 1589.2 cm^−1^, 1587.0 cm^−1^, 1418.2 cm^−1^, and 1407.3 cm^−1^ in both UAE and HW samples correspond to asymmetric and symmetric C=O stretching vibrations of carboxyl groups, confirming the presence of uronic acids (Chen, Wang, et al., 2023; Saleh et al., 2017). Stronger peaks in these regions were observed in the UAE sample compared with HW, suggesting a higher uronic acid content in UAE-extracted polysaccharides. The absorption region at 1000–1200 cm^−1^ was attributed to C—O—C glycosidic bond stretching vibrations and C—O—H side group vibrations, indicating the presence of pyranose rings (Huang et al., 2020; Yuan et al., 2020). A broader band in this region was observed for UAE polysaccharides, reflecting greater heterogeneity in the vibrational environment of C—O bonds (Wang et al., 2023). Absorption peaks near 870 cm^−1^ were detected in both samples and were assigned to characteristic β-glycosidic bond vibrations (Lin et al., 2023), indicating that the β-configuration of the polysaccharides was preserved during both extraction processes. Characteristic absorption bands of pyranose rings appeared in the range of 864.52–919.25 cm^−1^ (Du et al., 2003). UAE-extracted polysaccharides showed multiple well-resolved peaks in this region, whereas HW samples exhibited broader and less distinct peaks. These differences suggest greater structural heterogeneity in glycosidic linkage types and substitution patterns in UAE polysaccharides (Tang, Miao, et al., 2023). This may be attributed to more extensive disruption of cell wall structures under UAE conditions, leading to the release of polysaccharide fractions with more diverse structural features (Tang, Miao, et al., 2023).

### 3.11 Analysis of NMR

**Fig. 6:**
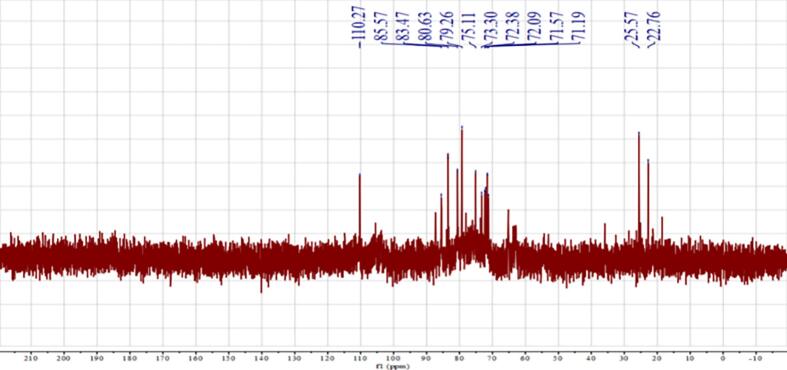
^13^C NMR spectrum of *N. commune* polysaccharides (UAE).

In the ^13^C NMR spectrum, anomeric carbon signals of polysaccharides obtained by UAE generally appear in the downfield region between 90 and 110 ppm. As shown in Fig. 6, a prominent signal was observed at 110.27 ppm. Previous studies have reported clear differences in the chemical shifts of α- and β-pyranose anomers in this region. α-Anomers typically resonate between 90 and 102 ppm, whereas β-anomers resonate between 102 and 112 ppm (Lin et al., 2023). Based on this, the main sugar residues in the polysaccharide sample are inferred to adopt a β-pyranose configuration. Unsubstituted C-2, C-3, and C-4 carbons generally resonate in the range of 70–77 ppm, while signals above 78 ppm indicate substitution at one or more of these positions. Unsubstituted C-6 carbons typically appear between 60 and 64 ppm and shift to 67–70 ppm upon substitution (Wang, Yu, et al., 2022). In this study, signals in the range of 71.19–79.26 ppm were attributed to 1,2 → and 1,4 → glycosidic linkages (Xia et al., 2009), while signals at 80.63–85.57 ppm suggested the presence of 1,3-linked sugar residues in the polysaccharide chain (Yuan et al., 2020). In addition, a signal at 22.76 ppm was assigned to methyl carbon. This indicates that the polysaccharide may contain *O*-methylated or *N*-methylated groups, or other structural moieties bearing methyl substituents (Wang et al., 2025). In summary, the polysaccharides are mainly composed of β-pyranose units. Multiple glycosidic linkage patterns, including 1,2-, 1,3-, and 1,4-linkages, as well as methylation modifications, were identified in the polysaccharide structure. However, further studies are still required to fully elucidate the detailed structure of these polysaccharides.

### 3.12 In vitro antioxidant activity of *N. commune* polysaccharides

#### 3.12.1 DPPH radical scavenging capacity

**Fig. 7:**
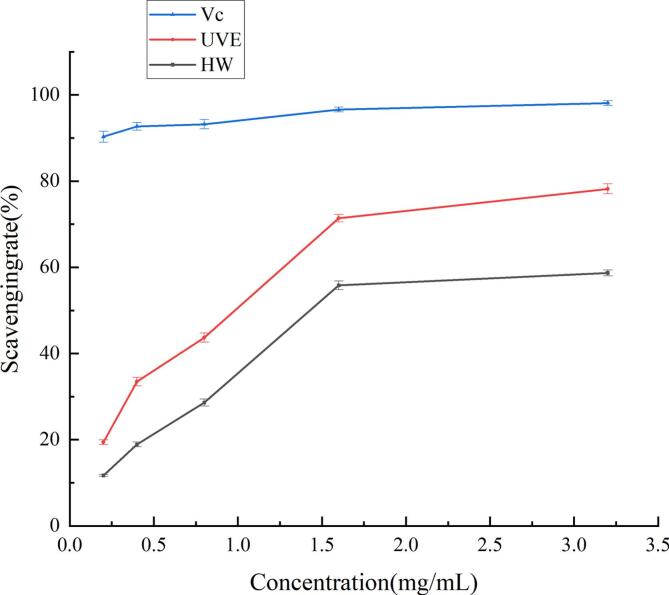
DPPH radical scavenging capacity.

As shown in Fig. 7, *N. commune* polysaccharides exhibited a clear dose-dependent DPPH radical scavenging activity over the concentration range of 0–3.2 mg/mL. UAE-derived polysaccharides showed significantly higher DPPH scavenging capacity than HW-derived polysaccharides at all tested concentrations. This difference is mainly attributed to the moderate depolymerization effect induced by ultrasonic treatment. High-molecular-weight polysaccharide chains are cleaved by the shear forces generated during ultrasonic cavitation, leading to the exposure of more reducing termini and active sites. Accordingly, the ability of polysaccharides to donate hydrogen atoms or electrons to DPPH radicals is enhanced. This result is consistent with the findings of Zhang et al. (Zhang, Chen, et al., 2025), who reported that ultrasonic degradation can improve the structure-activity relationship of fungal polysaccharides. In addition, monosaccharide composition analysis showed that the glucuronic acid content in UAE polysaccharides increased from 9.87% to 10.36%, which contributes to the improved DPPH scavenging activity. The carboxyl group (—COOH) in uronic acid residues can act as a hydrogen donor to react directly with free radicals. Moreover, deprotonated carboxylate anions further enhance antioxidant efficiency through resonance stabilization (He et al., 2016; Li et al., 2016). Quantitative structure-activity relationship studies have also confirmed that MW distribution and uronic acid content are two key structural parameters governing the DPPH radical scavenging activity of polysaccharides (Li et al., 2016). In summary, the UAE process enhances the DPPH radical scavenging activity of polysaccharides by optimizing MW distribution and increasing uronic acid content.

#### 3.12.2 ·OH scavenging capacity

**Fig. 8:**
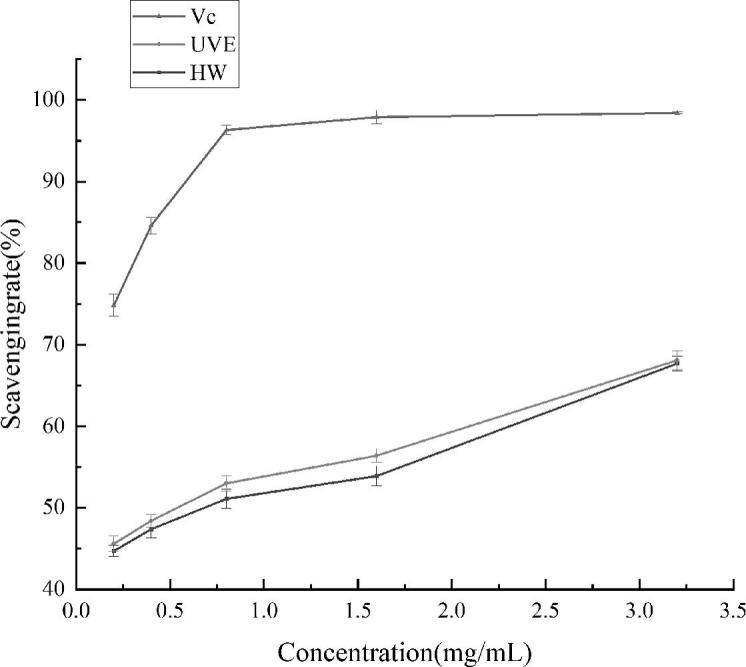
·OH scavenging capacity.

As shown in Fig. 8, the ·OH scavenging capacity of *N. commune* polysaccharides obtained by both extraction methods increased with increasing concentration over the range of 0–3.2 mg/mL, indicating a clear dose-dependent effect. However, no significant difference was observed between UAE- and HW-extracted samples. This may be attributed to the extremely high redox potential and reaction rate constant of the ·OH. Its scavenging process mainly occurs through non-specific electron transfer or hydrogen atom transfer involving broad-spectrum reducing groups in the system (Chen, Wei, et al., 2023; Su & Li, 2020b). The reaction is diffusion-controlled and is therefore less sensitive to subtle conformational differences or partial MW reduction of polysaccharide chains compared with the DPPH system. As a result, as long as a sufficient density of nucleophilic functional groups is maintained in the polysaccharide structure, both intact high-molecular-weight chains and partially depolymerized fragments can exhibit comparable ·OH scavenging activity. Nevertheless, low-molecular-weight polysaccharide fractions generated by UAE generally show higher water solubility and improved bioavailability, which may translate into greater practical antioxidant potential in physiological environments or during transmembrane transport processes (Wang, Yan, et al., 2022).

#### 3.12.3 O^2−^· scavenging capacity

**Fig. 9:**
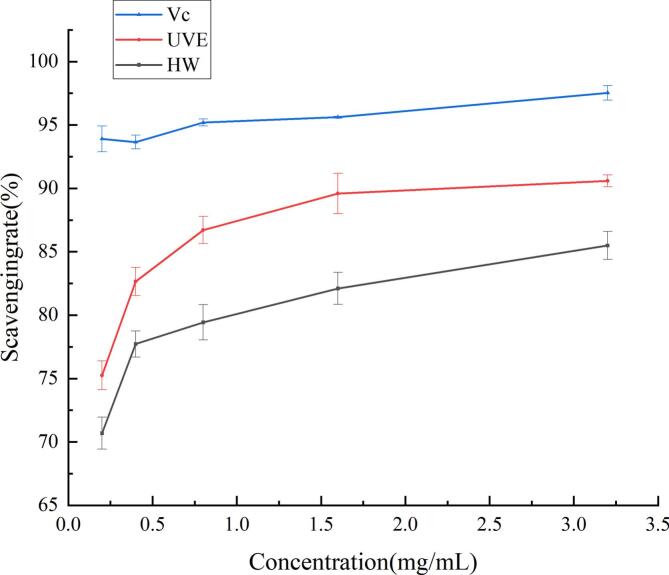
O^2−^· scavenging capacity.

As shown in Fig. 9, the O_2_^−^· scavenging activity of *N. commune* polysaccharides increases significantly with increasing concentration over the range of 0–3.2 mg/mL. UAE-derived polysaccharides exhibit higher scavenging activity than HW-derived polysaccharides. Superoxide anion is the primary radical in the cascade generation of endogenous reactive oxygen species, and its removal mainly occurs through electron transfer mechanisms. The UAE process leads to a marked reduction in the average MW of polysaccharides. After MW reduction, polysaccharide chains adopt more flexible conformations, and additional active groups, such as hydroxyl and carboxyl groups, are exposed. As a result, the collision frequency and mass transfer efficiency between electron donors and free radicals are enhanced. This finding is consistent with the results reported by Zhou et al. (Zhou et al., 2025). in their study on UAE of Pleurotus nebrodensis polysaccharides. They demonstrated that moderate MW reduction can significantly improve O₂^−^· scavenging activity, primarily due to the higher diffusion coefficient and faster reaction kinetics of low-molecular-weight fractions. In addition, the higher glucuronic acid content in UAE polysaccharides contributes synergistically to their antioxidant activity. The electron-withdrawing effect of carboxyl groups polarizes adjacent hydroxyl bonds, thereby facilitating hydrogen atom dissociation. Consequently, the superoxide anion scavenging activity is further enhanced through a hydrogen atom transfer mechanism (He et al., 2016; Su & Li, 2020b).

#### 3.12.4 Anti-lipid peroxidation capacity

**Fig. 10:**
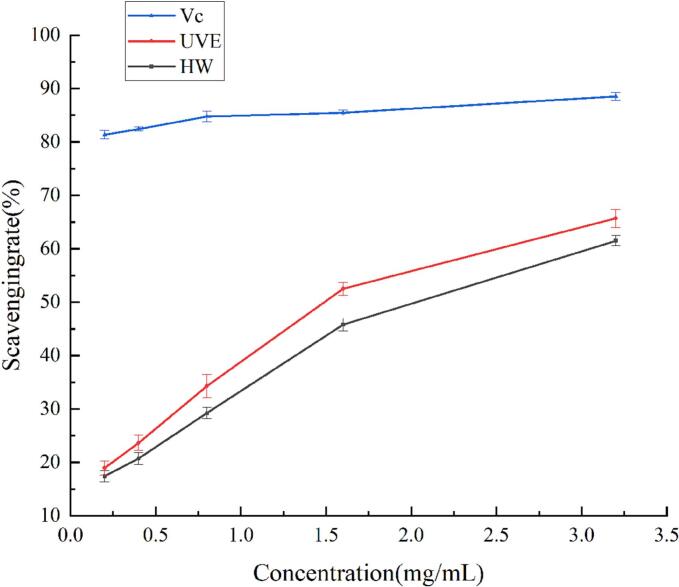
Anti-lipid peroxidation capacity.

As shown in Fig. 10, both *N. commune* polysaccharide samples exhibit concentration-dependent anti-lipid peroxidation activity over the range of 0–3.2 mg/mL, with UAE-derived polysaccharides showing stronger inhibitory effects. Lipid peroxidation is a free radical-initiated chain reaction. Polysaccharides can inhibit this process by chelating transition metal ions, scavenging lipid radicals, and forming physical barriers at the oil-water interface (Li et al., 2016). The enhanced anti-lipid peroxidation activity of UAE polysaccharides is likely associated with their improved interfacial adsorption capacity resulting from reduced molecular weight. Low-molecular-weight polysaccharide fragments exhibit lower steric hindrance and higher conformational flexibility. These properties facilitate their adsorption onto lecithin micelles or liposome surfaces, where they form dense hydrated protective films that inhibit the propagation of lipid peroxidation radicals. In contrast, high-molecular-weight polysaccharides tend to form less uniform and less compact interfacial layers due to greater steric hindrance, limiting their protective effectiveness (Su & Li, 2020b). In addition, the higher content of glucuronic acid residues in UAE extracts enhances their metal ion chelating ability. This effect suppresses metal ion-catalyzed Fenton reactions and effectively inhibits the initiation stage of lipid peroxidation chain reactions (Wang, Yan, et al., 2022; Zhou et al., 2025).

#### 3.12.5 Total reducing power

**Fig. 11:**
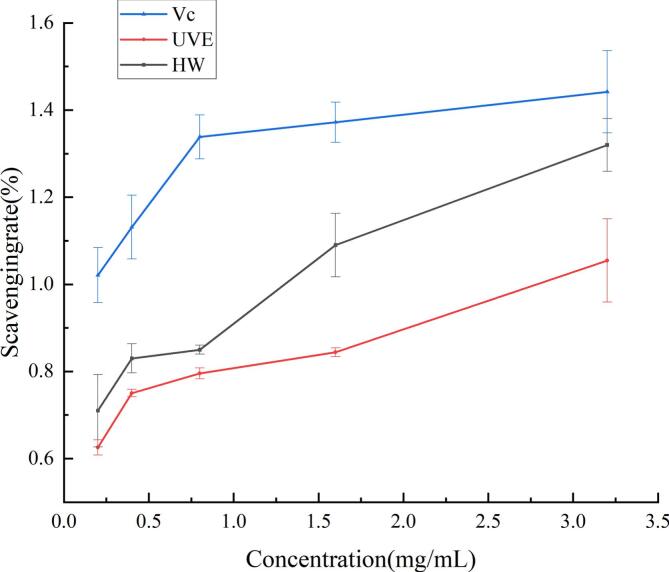
Total reducing power.

As shown in Fig. 11, the total reducing power of *N. commune* polysaccharides gradually increases with concentration from 0 to 3.2 mg/mL. At higher concentrations, UAE-derived polysaccharides exhibit stronger reducing capacity. The ferricyanide reduction system is used to evaluate the electron-donating ability of the samples. The higher reducing power observed for UAE polysaccharides suggests that their structural characteristics are more favorable for electron transfer reactions. Ultrasound-induced degradation of macromolecular chains increases the number of terminal reducing hemiacetal hydroxyl groups. It also exposes internal hydroxyl groups and active sites that are otherwise buried within high-molecular-weight folded structures. At higher concentrations, these exposed functional groups contribute synergistically to electron donation, thereby significantly enhancing the reducing potential of the system (Li et al., 2016; Zhang, Chen, et al., 2025). A negative correlation between polysaccharide reducing power and MW has been reported in the quantitative structure-activity relationship model proposed by Li et al. (Li et al., 2016) Low-molecular-weight polysaccharides generally show better performance in chemical reduction systems due to their larger specific surface area and higher density of active functional groups. In the present study, the MW of *N. commune* polysaccharides is reduced by the UAE method, and this reduced M_W_ range is considered favorable for efficient electron transfer in reduction systems (Wang, Yan, et al., 2022; Zhang, Chen, et al., 2025).

## 4 Conclusion

Based on the results of the single-factor experiments, a PB design was first applied to identify the key factors significantly affecting the UAE of *N. commune* polysaccharides. A steepest ascent design was then used to determine the optimal region for response surface analysis, followed by a BB design to optimize the extraction conditions. Under the optimized conditions, solid-to-liquid ratio of 1:80 (g/mL), enzyme dosage of 0.3 g, enzymolysis temperature of 60 °C, enzymolysis time of 60 min, ultrasound power of 780 W, ultrasound time of 40 min, and ultrasound temperature of 50 °C, the polysaccharide yield reached 34.11%. FT-IR analysis showed that the UAE method increased both polysaccharide yield and uronic acid content, while having minimal effect on the fundamental glycosidic bond structure. In addition, FT-IR and NMR analyses confirmed the presence of characteristic polysaccharide structural features. Further structural analysis revealed that UAE-derived polysaccharides exhibited lower molecular weights and a similar monosaccharide composition dominated by glucose, xylose, galactose, and glucuronic acid compared to HW. Comparative antioxidant assays further demonstrated that polysaccharides extracted via UAE exhibited significantly stronger antioxidant activity than those obtained by conventional HW.

## CRediT authorship contribution statement

**Jinhua Shao:** Writing – review & editing, Writing – original draft, Supervision, Resources, Project administration, Methodology, Conceptualization. **Chenxu Wu:** Validation, Investigation, Data curation. **Quan Li:** Validation, Investigation, Data curation. **Yuting He:** Project administration. **Zhiyong Zhu:** Visualization, Software, Formal analysis.

## Funding

This study received no external funding.

## Declaration of competing interest

The authors declare that they have no known competing financial interests or personal relationships that could have appeared to influence the work reported in this paper.

## Data Availability

All data generated or analyzed during this study are included in this published article.
